# Liquid biopsy for monitoring of tumor dormancy and early detection of disease recurrence in solid tumors

**DOI:** 10.1007/s10555-022-10075-x

**Published:** 2023-01-06

**Authors:** Isabel Heidrich, Benjamin Deitert, Stefan Werner, Klaus Pantel

**Affiliations:** 1 Institute for Tumor Biology, University Cancer Center Hamburg, University Medical Center Hamburg-Eppendorf, Martinistr. 52, 20246 Hamburg, Germany; 2 Skin Cancer Center, Department of Dermatology and Venereology, University Hospital Hamburg-Eppendorf, Hamburg, Germany; 3 Mildred-Scheel-Nachwuchszentrum HaTRiCs4, Universitäres Cancer Center Hamburg, Hamburg, Germany

**Keywords:** Minimal residual disease, Disseminated tumor cells, Circulating tumor cells, Circulating tumor DNA, Breast cancer, Prostate cancer, Melanoma

## Abstract

Cancer is one of the three leading causes of death worldwide. Even after successful therapy and achieving remission, the risk of relapse often remains. In this context, dormant residual cancer cells in secondary organs such as the bone marrow constitute the cellular reservoir from which late tumor recurrences arise. This dilemma leads the term of minimal residual disease, which reflects the presence of tumor cells disseminated from the primary lesion to distant organs in patients who lack any clinical or radiological signs of metastasis or residual tumor cells left behind after therapy that eventually lead to local recurrence. Disseminated tumor cells have the ability to survive in a dormant state following treatment and linger unrecognized for more than a decade before emerging as recurrent disease. They are able to breakup their dormant state and to readopt their proliferation under certain circumstances, which can finally lead to distant relapse and cancer-associated death. In recent years, extensive molecular and genetic characterization of disseminated tumor cells and blood-based biomarker has contributed significantly to our understanding of the frequency and prevalence of tumor dormancy. In this article, we describe the clinical relevance of disseminated tumor cells and highlight how latest advances in different liquid biopsy approaches can be used to detect, characterize, and monitor minimal residual disease in breast cancer, prostate cancer, and melanoma patients.

## Identifying regulators and biomarker of tumor dormancy using liquid biopsy

**Fig. 1:**
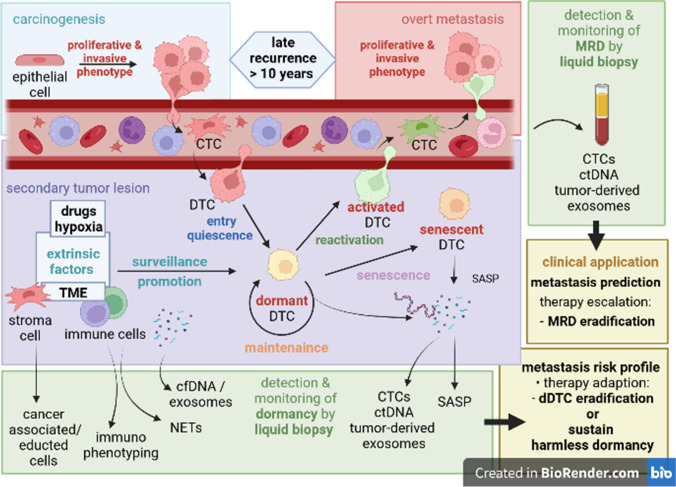
Dormancy. Dissemination and tumor dormancy—MRD and dormancy biomarkers indicating late tumor recurrence. TME tumor microenvironment, CTC circulating tumor cell, ctDNA circulating tumor DNA, MRD minimal residual disease, NETs neutrophil extracellular traps, SASP senescence-associated secretory phenotype, DTC disseminated tumor cell, dDTC dormant disseminated tumor cell. During carcinogenesis, tumor cells undergo different adaptions enabling malignancies to invasive growth, escape of immune surveillance, epithelial–mesenchymal transition, and induction of tumor vascularization. Often, intravasation and dissemination were achieved before solid malignancies were clinically captured or have been detected by radiographic diagnostics. Minimal residual disease (MRD) tracking with liquid biopsy might be a more suitable diagnostic instrument for detection of early blood dissemination. Enhanced by metastatic niche preparation, disseminated tumor cells (DTC) extravasate and colonize secondary organs and form immediately overt metastasis. However, some entities tent to endure in a tumor dormant status. Previously invasive and proliferative tumor cells switch therefore into a quiescent cell state in which these cells resist immunity for years. Driven by a plethora of partly unknown processes and programs, dormant disseminated tumor cells (dDTCs) awake and re-gain proliferative phenotypes. For early prediction of disease progression, there is an urgent need for the exploration and clinical introduction of blood-based liquid biopsy biomarkers indicating early dynamics in MRD. On the other side, detection of dormant DTCs resting in cell cycle arrest and in metabolic quiescence circumventing invasive procedures like bone marrow punctation might be challenging and requires complementary and comprehensive tumor-derived and microenvironment biomarkers

Tumor dormancy circumscribes different modalities of quiescence and constant tumor load. Tumor mass dormancy on the one side expresses an equilibrium state of tumor cell apoptosis and proliferation from a holistic point of view. This steady state finds it clinical equivalent in the term of minimal residual disease (MRD), whereas recently also the term measurable residual disease has been proposed for leukemia, which might also become appropriate for solid tumors [1]. MRD denotes the cellular networks of gene regulation, cell signaling, and metabolic reactions, shaping dormant states. In contrast to mass dormancy, cellular dormancy aims to elucidate molecular profiles of singular disseminated tumor cells. As described by Sosa, Bragado, and Aguirre-Ghiso [2], cellular dormancy regulated by autogenously programs and tumor microenvironmental drivers have immediate impact on tumor mass dormancy and therefore on MRD (Fig. 1). Vice versa, a profound understanding of the biological mechanism driving tumor dissemination and controlling quiescence of disseminated tumor cells (DTCs) are of emergent clinical interest. Blood-based, non-invasive liquid biopsy biomarkers monitored by clinicians open perspectives for therapeutics maintaining tumors dormant or eradicate DTC [3].

By the event of an organ donation harboring undetected MRD from a long-term cancer survivor, a metastatic outbreak in an immune deficit patient has been observed [4]. This case report has first led to the principle of immune surveillance, postulating the predominant role of immunity in controlling MRD [5, 6]. Secondly, the MRD outbreak has shown dissemination of local disease earlier than previously expected. More evidence—in line with this case report—has emphasized the notion of early dissemination in malignant disease [7–10] with implications for therapy strategies and, respectively, for implementation of sensitive blood-based biomarkers indicating early dissemination. To date, multiple quiescence drivers (e.g., SOX9 or SOX2) have been identified, reactivating progenitor stem cell programs, and are simultaneously known for their function in immune tolerance and therapy resistance [11]. However, quiescent cells with key features like autophagy, G0 cell cycle arrest, and immune surveillance might be difficult to detect in cancer patients. This dilemma can be circumvented by the principle of liquid biopsy, first described by Pantel and Panabieres [12], which allows the specific analysis of tumor markers such as circulating tumor cells (CTC), circulating tumor DNA (ctDNA) by simple and non-invasive blood sampling or by the direct detection of DTCs in secondary organs like the bone marrow (BM).

Cancer cell dormancy is embedded in the cascade of metastasis formation. When precancerous lesions overcome self-protection programs like senescence by mutational or epigenetic loss of tumor suppressors and gain of oncogenic drivers, processes predisposing facilitate motility of tumor cells. Early within carcinogenesis, malignant cells managed under selection pressure to gain the ability to influence and modify their tumor microenvironment [13, 14]. Cancer cells escape epithelial cell formation by epithelial–mesenchymal transition (EMT) to invade into their stromal adjacent tissue (invasion) and become CTCs. Thereby, cancers induce neoangiogenesis, predominate immune surveillance by inducing immune tolerance, and enhance invasiveness [13]. In the bloodstream, CTC face new challenging environmental circumstances and therefore just a minority of CTCs resist and invade into their secondary lesions, such as the liver, BM, or lungs, and become DTCs [15].

Extrinsic mechanisms and tumor cellular dormancy are mutually related [16]. Adaptive immunity with crucial function for tumor mass dormancy impairs and control the tumor in its phenotypical appearance. A recent work promotes the idea of close relation of tumor intrinsic modification reducing cell cycle dynamics and facilitate immune evasion allowing long-term dormancy in distant metastatic niches [17]. In MRD patients, the presence of mutant cells that are primarily resistant to the applied anti-cancer drugs or the presence of tumor cells that become secondarily drug resistant due to activation of survival pathways during therapy is frequently observed. Thus, another reason for the establishment of tumor dormancy is the development of therapy resistance in individual cancer cell clones [18]. In this context, therapy-induced senescence has long been recognized as an important mechanism that enables tumor cells to escape the direct impact of a cytotoxic therapy by enabling cell survival in a dormant state [19]. The senescence-associated secretory phenotype as well as the reversion of the senescent state can contribute to disease recurrence and escape from tumor dormancy [19–21]. Recent findings demonstrate that treatment itself can also actively induce tumor dormancy through a diapause-like adaptation, which is a reversible state of suspended embryonic development activated by hostile environmental conditions [22, 23].

Over the past decade, a growing body of research suggests that malignancies adapt to selection pressure as therapies like immune checkpoint inhibitors [24] or targeted therapies [25] or to hypoxia [26, 27] with similar adaptations rendering quiescence a clinically pivotal biomarker target. Besides clinical applications, investigation of CTC, ctDNA, and DTCs might give new impulses for the biologic procedure of dissemination. Nevertheless, the detection and analysis of these biomarkers need to overcome major clinical and technical challenges and thus require several sensitive methodologies, which are discussed in the following chapter.

## Methodology and technical challenges of liquid biopsy

**Fig. 2:**
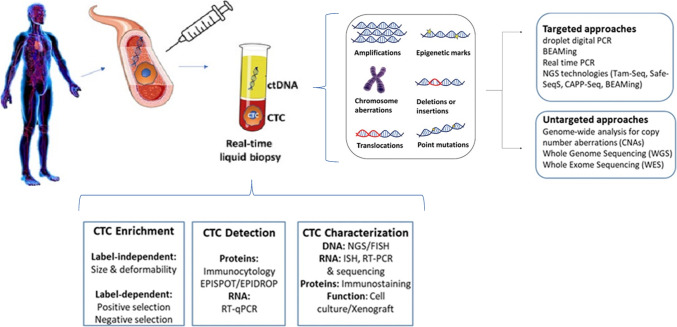
Methods. Technologies for enrichment, detection, and characterization of circulating tumor cells (CTCs) and circulating DNA (ctDNA) detection technologies. CTCs isolated from blood samples can be enriched using marker-dependent techniques. After enrichment, the isolated CTCs can be identified using immunocytological assays. The functional properties of CTCs can also be investigated in vivo by the establishment of CTC-derived xenografts. ctDNA detection technologies: ctDNA analysis is based on the identification of tumor-specific aberrations or epigenetic marks in cfDNA samples. Ultrasensitive targeted approaches allow fast, cheap, and sensitive detection of mutations. Untargeted approaches allow the unbiased detection of genomic aberrations without requiring prespecified information about the mutation pattern of the respective primary tumor. Source: modified from Heidrich et al., Int. J. Cancer. 2021; https://doi.org/10.1002/ijc.33217

Only a small subpopulation of CTCs released from primary or metastatic lesions is able to survive in blood for a short transit time, due to an immense stress exposure, which, in part, explains the low concentration of CTCs in blood samples from cancer patients and requirement to use ultrasensitive methods for the enrichment and detection methods of these rare cells [28]. CTCs can be enriched by physical criteria that distinguish them from normal blood cells such as size, deformability, or electrical charge of the cell membrane (Fig. 2). These technologies do not depend on the expression of a tumor marker antigen and are therefore denoted as “label-independent” (e.g., Hydro-Sec and CTC-iChip) [29–32]. Alternatively, label-dependent technologies are applied that are either based on positive or negative selection of CTCs. Positive enrichment methods use cell surface markers frequently expressed on tumor cells and absent or rarely expressed on normal blood cells (e.g., EpCAM, Mucin-1, HER2, or EGFR). Negative enrichment is based on the removal of normal blood cells by antibodies against CD45 or other antigens expressed on leukocytes or circulating endothelial cells [32, 33]. After enrichment, the isolated CTCs can be identified using immune–cytological assays like membrane and/or intracytoplasmic staining with antibodies to epithelial, mesenchymal, tissue-specific, or tumor-associated markers (e.g., keratins). Because BM is a mesenchymal organ and DTCs from most other solid tumors are derived from epithelial organs, this principle is also applied for the detection of DTCs in BM samples [34]. Molecular assays enable the identification of CTCs at the DNA, RNA, and protein level. Besides immunocytological approaches, secretion of tumor-associated proteins can be used to enumerate viable CTCs using the EPISPOT or EPIDROP technologies [35] that enables the detection of single CTCs in microdroplets. The functional properties of CTCs can also be investigated in vivo by the establishment of CTC-derived xenografts. Recent studies using xenografts models of CTC cell lines allowed first insights into functional properties and response to drugs of CTCs [28, 36].

Cell-free DNA (cfDNA) circulating in the peripheral blood is mostly released through necrosis and apoptosis but potentially also by secretion through extracellular vesicles. In cancer patients, only a small portion of cfDNA (usually 0.01–5%) is ctDNA shed into the blood by tumor cells [37]. ctDNA analysis requires the use of ultrasensitive methods based on the identification of tumor-specific aberrations or epigenetic marks in cfDNA samples [38] (Fig. 2). Ultrasensitive targeted approaches like droplet digital PCR or BEAMing and NGS technologies (Tam-Seq, Safe-SeqS, and CAPP-Seq) are able to detect prespecified cancer-associated mutations at high sensitivity. Refined real-time PCR methods, like allele-specific PCR (AS-PCR), allele-specific non-extendable primer blocker PCR (AS-NEPB-PCR), co-amplification at lower denaturation temperature (COLD-PCR), or peptide nuclei acid-locked nucleic acid (PNA-LNA) PCR clamp allow fast, cheap, and sensitive detection of mutations. Untargeted approaches like whole-genome sequencing, whole-exome sequencing, or FastSeqS allow the unbiased detection of genomic aberrations without requiring prespecified information about the mutation pattern of the respective primary tumor [39, 40]. Even though targeted approaches show high analytical sensitivity, they are limited to mutations in a set of predefined genes, whereas untargeted approaches like whole-genome sequencing or whole-exome sequencing provide the opportunity to detect novel, clinically relevant genomic aberrations without requiring information about the primary tumor [41]. It is essential to avoid conditions that increase non-tumorous cfDNA in the presence of small amounts of ctDNA, since most cfDNA originates from normal cells. Besides fast processing of the sample, ambient temperatures, double plasma centrifugation, and special cfDNA blood collection tubes, parallel sequencing of normal leukocytes is required to differentiate clonal hematopoiesis of indeterminate potential mutations from somatic tumor mutations [42–44]. In this review, we will focus on describing the clinical relevance of MRD as well as the role of liquid biopsy (ctDNA, CTCs, und DTCs) for detection, monitoring, and prediction of recurrence. We place emphasis on studies with breast cancer, prostate cancer, and malignant melanoma patients. Breast and prostate cancer belong to the most frequent tumor entities, and melanoma has become the prime target for immunotherapies using liquid biopsy to assess treatment responses.

## Breast cancer

### Clinical relevance of MRD in patients with breast cancer

Breast cancer is the most commonly diagnosed cancer in women and the main cause of cancer-related death for women worldwide [45]. In breast cancer, 70–80% of patients harbor primary tumors expressing hormone receptors for estrogen as major growth stimulus [46, 47]. These tumors can be well-treated by drugs targeting the ER-signaling axis with remarkable 5-year survival rates of over 90% [47–49]. However, around 20% of patients relapse and develop recurrence after the 5-year surveillance period [50–52]. Early recurrence is mainly defined by events of metastasis 3 to 5 years after first diagnosis and is closely related to an aggressive, therapy-resistant tumor [53]. However, analysis of 20-year follow-up has shown that ER-positive patients show continuously late relapses [52] most likely due to MRD or dormant DTCs that become activated by so far unknown extrinsic and intrinsic stimuli. Interestingly, approximately 50% of breast cancer patients with DTCs in their BM show no tumor recurrence within 10 years observation time [34]. Estimation of the total tumor load of DTCs in BM from these data results in the astonishing conclusion that these patients are able to control the outgrowth of at several hundred thousand of tumor cells in BM. If one assumes that the BM is not the only reservoir for DTCs, this number is probably even much higher. Until to date, metastatic disease is a non-curable state of breast cancer, underlining the clinical importance of intervention for eradication or control of MRD to prevent metastatic outbreak.

To prevent late recurrence, a prolonged endocrine standard of care therapy might suppress MRD long lastingly and can prevent thereby disease outgrowth [54–56]. Although, endocrine therapy is a well-tolerated, cost-efficient therapy option, subgroups under low recurrence risk prone to overtreatment and patients under high risk would benefit from an enhanced and multimodal systemic therapy. Currently, there are several trials aiming to target MRD with systemic adjuvant treatment options including liquid biopsy analyses for therapy stratification and monitoring (NCT04523857, NCT00429247, and NCT01779050).

### Monitoring MRD in breast cancer patients by detection and characterization of DTCs

For screening and monitoring of MRD, DTC characterization from BM aspirates were introduced and appear as additional micrometastasis searching tool besides the sentinel lymph node investigation [57]. BM aspiration is an invasive method but procedure integration into the surgical setting with general anesthetics can provide a pain-reduced opportunity for gaining information about DTCs. If BM aspirate analysis precise the prognosis prediction in comparison to clinicopathological criteria like tumor size, nodal status, tumor grading, and molecular subclassification, this investigation would be legitimate for incorporation into clinical routine. Previously, Braun et al. showed in a multicenter study that DTC detection in BM independently predicted prognostic values [34]. This finding has been more recently confirmed in a large-scale single-center investigation on 803 patients out of 3141 patients, showing that DTC positivity was an independent prognostic marker for disease-free survival (DFS) and overall survival (OS) [58]. By finding 2814 patients with detectable DTC from a 10,307 patient’s cohort, a multicenter international study could further strengthen the independence as prognostic marker [59]. Interestingly, DTC detection appears to provide information independent from the recurrence score determined by the analysis of primary breast tumor tissue like the Oncotype DX score [60].

Besides DTC enumeration, the additional molecular characterization of DTCs at the single-cell level can provide deeper insights into the biology of MRD. This might hold the potential for identifying biological key signatures as targets for adjuvant therapies for each patient’s MRD and can personalize adjuvant therapy that target those signatures. Interestingly, DTCs isolated from BM appears to show HER2 amplification in patients with HER2-negative primary tumors [61–63]. Accordingly, HER2 status of DTCs can offer one opportunity to eradicate MRD, as it was shown in previous work [64] and is under current investigation in trials (NCT00429247 and NCT01779050). Besides HER2, primary breast cancer tissue and DTCs can also differ in terms of their PIK3CA mutations, EPCAM upregulation, MYC and CCNE1 oncogene upregulation, or ESR1 expression, underlining the heterogeneity of DTCs [65]. Whether dormant DTCs harbor cancer-stem cell characteristics remains subject of ongoing investigations. Balic et al. showed that 72% of breast cancer patients analyzed harbored DTCs with a CD44 + /CD24–stem cell phenotype [66]. Whether or whether not these stem-cell like DTCs are able to form metastasis remains to be demonstrated. Whole-genome amplification followed by DNA analysis for copy number aberrations or mutations [8] might provide more comprehensive information on the genomic background of DTCs, and the comparison to overt metastases might allow the identification of metastasis-initiating cells.

### Monitoring MRD in breast cancer patients using blood-based biomarker

#### Circulating tumor cells (CTCs)

Analysis of DTCs in BM (or other organs) is invasive and thus difficult to repeat. In contrast, peripheral blood can be easily and repeatedly obtained by a simple venous puncture and has therefore become the preferred fluid for liquid biopsy analyses [3]. CTCs in the blood circulation are cells that originate from primary and (micro-) metastatic lesions. Despite the assumption that only a small fraction of CTCs will develop into metastasis [67], the CTC counts at initial diagnosis and during the post-surgical follow up period are tightly correlated to the risk of relapse in breast cancer and other solid tumors [32]. Dissemination of tumor cells through the blood circulation is therefore an important intermediate step that also exemplifies the switch from localized to systemic disease [15] and recent mathematical modeling revealed that the survival of CTCs during their dangerous passage though the blood might be even the largely underestimated key step in cancer metastasis [68].

The detection and molecular characterization of CTCs may provide important insights into the biology behind metastatic progression. Due to the insufficient performance of serum markers (CA 15–3 or CEA) regarding sensitivity and the lack of proof of a survival advantage using protein-based biomarker [69–71], other blood-based tests are desperately required. The clinical significance of CTCs has been extensively evaluated in patients with breast cancer, demonstrating that CTC detection is associated with OS and PFS both in early and metastatic breast cancer. In MRD stages of breast cancer without overt metastatic lesions, CTC numbers are low, and sufficient blood volumes as well as sensitive assays are required for detection (e.g., up to 20 mL of blood) [72]. Various studies have demonstrated that the detection of CTCs at initial diagnosis is correlated with an increased risk for metastatic relapse, suggesting that tumors with a higher propensity to release malignant cells into the circulation have a higher chance to eventually form overt metastases at distant organs [72–75]. These findings may change the current risk assessment of early breast cancer because they clearly indicate the metastatic potential of CTCs early in the disease. In the 2018 Tumor staging Manual of the American Joint Committee on Cancer (AJCC), a new risk category called cM0(i +) was introduced. However, CTC testing has not been implemented into clinical practice yet.

Less information is available regarding the prognostic relevance of liquid biopsies focusing on the surveillance of MRD through follow-up care studies. Here, we discuss recent studies indicating that the detection of CTC months or even years after initial diagnosis predicts metastatic relapse earlier than clinical imaging procedures used to diagnose relapse in breast cancer patients. Trapp et al. assessed the CTC counts before and two years after chemotherapy in patients with non-metastatic breast cancer [76]. Two years after chemotherapy, 198 (18.2%) of 1087 patients were CTC positive, and a positive CTC status at this time point predicted a decreased OS and DFS. Similarly, Sparano et al. demonstrated that the presence of CTCs obtained approximately 5 years after diagnosis predicted late recurrence of patients with operable human epidermal growth factor receptor 2-negative breast cancer [77]. The analysis of 547 women revealed that the recurrence rates per person-year of follow-up in the CTC-positive group was 21.4% (7 recurrences per 32.7 person-years), while being only 2.0% in the CTC-negative group (16 recurrences per 796.3 person-years). Multivariate analysis showed that the detection of CTCs was linked to a 13.1-fold higher risk of recurrence (hazard ratio point estimate, 13.1; 95% CI, 4.7–36.3), thus indicating the clinical validity of CTCs for detection of MRD and risk stratification concerning late breast cancer recurrences. In view of the transit time of CTCs in the circulation, CTCs in patients detected years after removal of the primary tumor are most likely derived from occult micrometastatic lesions missed by radiological imaging or even single DTCs that extravasate back into the blood from their secondary site. Evidence for such a re-circulation of metastatic cells is derived from experimental models [78]. In addition, breast cancer patients with overt metastatic relapse have usually higher CTC counts than early stages patients, suggesting that CTCs are frequently released from metastatic lesions into the blood. To which extent these CTCs contribute to metastatic progression from single sites (“oligo-metastasis”) to multiple sites is still subject of investigation. Recently, it has been also suggested that the release of CTCs is following a circadian rhythm [79, 80].

#### Circulating tumor DNA (ctDNA)

The detection of ctDNA shedded by micrometastatic lesions also offers the opportunity for monitoring MRD [3]. Currently, ctDNA detection assigns patients into intervention trials aiming to eradicate MRD and imped metastasis outgrowth (NCT04985266, NCT04567420, NCT03285412, and NCT04915755), and it is used as surrogate endpoint in trials to anticipate response indicated by clearance of ctDNA by therapy (NCT03285412 and NCT03145961) (Table 1). In the recent ESMO guidelines [81], ctDNA detection for MRD detection is, however, not yet recommended for clinical practice. Assays need to be very sensitive to detect the minute amounts of ctDNA fragments in patients with MRD and assay harmonization and independent technical validation is urgently needed. In addition, the results of interventional clinical trials are needed to demonstrate the clinical utility of early recurrence detection by ctDNA. Although it is well accepted that ctDNA surveillance of blood samples leads to early detection of recurrence many months before radiological imaging (Prediction of metastatic growth already 2 years before relapse) [82], the clinical consequence of this finding and its benefit for prolonging the life span of cancer patients needs to be demonstrated before implementation in clinical practice.

**Table 1:** A selection of clinical trials/studies on the implementation of liquid biopsy into clinical practice in breast cancer, prostate cancer and melanoma

Breast cancer					
Study/trial (year)	Inclusion criteria	*n* Pat	Methods/design	Clinical relevance	Ref
DARE (ongoing) Randomized phase II trial of ctDNA-guided second-line adjuvant therapy	High residual risk, Stage II-III, hormone receptor positive, HER2 negative	100	Signatera assay Palbocilcib and Fulvestrant are tested against standard of care endocine therapy	Primary outcome of ctDNA screening consists in ctDNA baseline incidence and prevalence of high-risk patients under standard of care. Secondary, correlation of ctDNA incidence and ouvert metastasis, association of ctDNA clearance to PFS and OS. Recurrence- free survival as additional endpoint	NCT04567420
TRAK TN (ongoing) Randomized trial utilizing ctDNA mutation tracking to detect MRD and trigger intervention in pat. with moderate and high-risk early stage triple neg	Early-stage, irrespective of hormone receptor and ERBB2 status, receiving neoadjuvant CTX followed by surgery or surgery before adj. CTX	314	Digital PCR Serial plasma samples taken every 3 months for the first year of follow-up and subsequently every 6 months	ctDNA detection during follow-up was associated with relapse (hazard ratio, 25.2; 95% CI, 6.7–95.6; *P* < 0.001). Detection of ctDNA at diagnosis, before any treatment, was also associated with relapse-free survival (hazard ratio, 5.8; 95% CI, 1.2–27.1; *P* = 0.01). In the combined cohort, ctDNA detection had a median lead time of 10.7 months (95% CI, 8.1–19.1 months) compared with clinical relapse and was associated with relapse in all breast cancer subtypes. Distant extracranial metastatic relapse was detected by ctDNA in 22 of 23 patients (96%)	[84]
SUCCESS A (2019) Multicenter, open-label, phase III trial compared two adjuvant CTX regimens followed by 2 vs 5 years of zoledronate	Early-stage, high-risk breast cancer patients	1087	ICC (CellSearch)	CTC status 2 years after chemotherapy had statistically significant prognostic relevance for OS (HR 3.91; 95% CI 2.04–7.52; *P* < 0.001) and DFS (HR 2.31; 95% CI 1.50–3.55; *P* < 0.001)	[76]
Papadaki et al. (2019) Investigation of the prognostic relevance of single CSC^+^/partial-EMT^+^ CTCs in patients with metastatic breast cancer and the effect of first-line chemotherapy on their incidence	Stage IV breast cancer	130	Triple immunofluorescence (keratins, TWIST, ALDH1)	CTCs that co-express keratin, high ALDH1 levels and nuclear TWIST1 correlated with reduced PFS (median 10.2 (8.9–11.6) vs 13.5 (11.3–15.7) months; *P* = 0.024). In HER2-negative patients, CSC + /partial EMT + CTCs were additionally associated with OS (median 39 (26.2–51.9) vs. 51 (15.7–86.4) months; *P* = 0.020)	[161]
Prostate cancer					
Study/trial (year)	Inclusion criteria	*n* Pat	Methods/design	Clinical relevance	Ref
Chen et al. (2019)	Metastatic and early PC (all stages)	54	ICC (CanPatrol platform, multi-RNA-ISH)	Positive rate of PGK1^+^/G6PD^+^ CTCs was higher in metastatic patients than in nonmetastatic patients (51.7% vs 8.0% (*P* = 0.002)). Hybrid CTCs were associated with Gleason score, tumor stage, tPSA level and cancer metastasis (*P* < 0.05). PGK1^+^/G6PD^+^ CTC number was correlated with the number of hybrid CTCs (*r* = 0.807; *P* < 0.001)	[114]
Budna-Tukan and Swierczewska et al. (2019)	High-risk PC (PSA ≥ 20 ng/mL and/or Gleason score on biopsy ≥ 8 and/or clinical tumor stage ≥ 2c), before and after RT	68	Antibody-based assays (CellSearch, CellCollector, EPISPOT Assay)	No difference in matched-pair analysis of CTC counts before and after RT for all three assays (CellSearch *P* = 0.28, dual fluoro-EPISPOT *P* = 0.27, CellCollector *P* = .36)	[116]
Haldrup et al. (2018)	PC before transurethral resections of the prostate or radical prostatectomy		Gene panel: *ST6GALNAC3*, *CCDC181*, *HAPLN3*	7% sensitivity and 100% specificity for patients with PC compared to patients with BPH (AUC = 0.833), combination improved sensitivity over best performing single marker (*HAPLN3*)	[122]
Study/trial (year)	Inclusion criteria	*n* Pat	Methods/design	Clinical relevance	Ref
Melanoma					
SECOMBIT (2021)	Stage III (unresectable) or stage IV melanoma with the BRAF V600 mutation	251	A three arms prospective, randomized phase II study to evaluate the best sequential approach with combo immunotherapy (ipilimumab/nivolumab) and combo target therapy (encorafenib/binimetinib)	*Sandwich approach* starting with targeted therapy for 8 weeks, then immunotherapy for 8 weeks; upon disease progression, following more targeted treatment showed improved progression-free survival of 15.1 vs. 10.6 months with vemurafenib/cobimetinib alone (*P* = 0.025). The OS rate at 2 and 3 years showed a better trend in arm B (immunotherapycombination) and C (sandwich approach)	[162]
COMBI AD (2020)	High risk, resected stage III melanoma with BRAF V600E or V600K mutations	870	A Phase III randomized double blind study of dabrafenib (GSK2118436) in combination with trametinib vs. two placebos in the adjuvant treatment after surgical resection	At 5 years, the percentage of patients who were alive without relapse was 52% (95% confidence interval (CI), 48 to 58) with dabrafenib plus trametinib and 36% (95% CI, 32 to 41) with placebo (hazard ratio for relapse or death, 0.51; 95% CI, 0.42 to 0.61). The percentage of patients who were alive without distant metastasis was 65% (95% CI, 61 to 71) with dabrafenib plus trametinib and 54% (95% CI, 49 to 60) with placebo (hazard ratio for distant metastasis or death, 0.55; 95% CI, 0.44 to 0.70)	[163]
Tan et al. (2019)	Resected stage III	133	Digital droplet PCR	ctDNA detection predicted patients at high risk of relapse at baseline (relapse-free survival (RFS) hazard ratio (HR) 2.9; 95% confidence interval (CI) 1.5–5.6; *P* = 0.002) and postoperatively (HR 10; 95% CI 4.3–24; *P* < 0.001). ctDNA detection at baseline (HR 2.9; 95% CI 1.3–5.7; *P* = 0.003 and postoperatively (HR 11; 95% CI 4.3–27; *P* < 0.001) was also associated with inferior distant metastasis-free survival (DMFS)	[151]
Lee et al. (2019)	IV	76	Digital droplet PCR	Longitudinal assessment of ctDNA in metastatic melanoma patients receiving treatment with PD1 inhibitors is an accurate predictor of tumor response, PFS and OS. Patients who had a persistently elevated ctDNA on therapy had a poor prognosis with a median OS 9.2 months (HR 0.02; *P* < 0.001), and this may guide combination and sequencing of subsequent therapies	[152]

Abbreviations: *AUC*, area under the curve; *BCSS*, breast cancer-specific survival; *BPH*, benign prostatic hypertrophy; *CI*, confidence-interval; *CTC*, circulating tumor cell; *ctDNA*, circulating tumor; *CTX*, chemotherapy; *ddPCR*, droplet digital PCR; *DDFS*, distant disease-free survival; *DFI*, disease-free interval; *DFS*, disease-free survival; *ER*, estrogen receptor; *HR*, hazard ratio; *ICC*, immunocytochemistry; *ITT*, intention to treat; *LRFS*, locoregional relapse-free survival; *NAT*, neoadjuvant therapy; *NCDB*, National Cancer Database; *OS*, overall survival; *Pat*., patients; *PC*, prostate cancer; *PR*, progesterone receptor; *RT*, radiation therapy; *TNBC*, triplet-negative breast cancer; *TR*, time ratio

Following the landmark study of Dawson and colleges [83], a plethora of studies have been published on the use of ctDNA. Interestingly, high-depth targeted capture massively parallel sequencing from primary tumor, residual tumors after chemotherapy, plasma samples with identified MRD and metastasis biopsies represented subclonal and clonal dynamics: whereas in only one patient congruence between primary tumor and MRD were identified, all other 4 patients developed incongruent prerelapsed MRD. Moreover, mutations that were found in MRD reoccurred in metastasis biopsies, revealing MRD as more similar to the distant metastasis then to their originating primary tumor [84]. Due to clonal and subclonal evolution tracking of primary lesion over MRD to overt metastasis, this evidence emphasizes the notion of the enrichment of diversity and acquiring of mutations in micrometastatic disease. Thus, MRD detection based on panels constructed by sequencing data from the primary tumor, which is the common approach for tumor-informed ctDNA assays, might be impeded by mutational diversity acquisition over time.

## Prostate cancer

### Clinical relevance of MRD in patients with prostate cancer

Prostate cancer is the most frequent malignancy in men in Europe and the USA. Most prostate cancer cases are detected when the primary tumor growth is still limited within the prostate [85]. Additionally, newly diagnosed patients in the Western countries have comparatively high 5-year survival rates and thus prostate cancer is considered as relatively slowly growing cancer type [85]. However, after a perceived curative therapy without detectable tumor, a recurrence can occur in about a quarter of the patients within the first five years after the initial cancer treatment [86]. The ability of prostate carcinoma cells to resume proliferation after a longer latency period and to initiate tumor recurrence is principally comparable to the biology of estrogen receptor-positive tumor cells [47].

Although breast and prostate cancer develop from organs of different anatomy and physiological function, both cancer entities follow common principles. As such, transformed epithelial cells from both organs require the steroid hormones estrogen or androgen to maintain cell proliferation in hormone-dependent cancer [87]. In contrast to breast cancer, prostate cancer patients are tested for their prostate-specific antigen (PSA) serum levels, which is a well-established marker for response and relapse monitoring [88]. This biomarker is unique for this cancer entity and exemplifies the advantages of the liquid biopsy concept, especially by enabling low-risk longitudinal measurements. Nevertheless, patients may not have an abnormal PSA value after the removal of the primary tumor [86], so it can be assumed that PSA measurement is not always appropriate to detect MRD in prostate cancer patients. However, a characteristic of prostate and breast cancer is the high propensity to metastasize to bone [89, 90]. For that reason, in both malignancies, the detection of DTCs in the BM has been widely used as indicator for MRD and source of metastatic relapse [91]. Detection of DTCs in the BM or detection of CTCs in the blood of prostate cancer patients are biomarkers that can be used to increase the precision of prognosis, and to monitor minimal residual cancer in an individual prostate cancer patient, which we discuss in the following chapter.

### Monitoring MRD in prostate cancer patients by detection and characterization of DTCs

Pioneering studies showed that the presence of epithelial-like cells in BM of prostate cancer patients might be interpreted as an indicator of the metastatic capacity of an individual primary tumor [92–94]. The detection of occult cells, positive for epithelial keratin expression, showed a significant correlation with established risk factors, such as local tumor extent, distant metastases, and tumor differentiation [95]. This indicated that BM is an important distant site for detecting early hematogenous spread of prostate carcinoma cells. Furthermore, immune–cytochemical detection of these cells may, therefore, be useful for increasing the precision of current tumor staging, and to monitor minimal residual cancer in an individual patient [95] especially in patients with clinically localized (T1-3N0M0) prostate carcinomas [96]. However, at that time point, it has not been established unequivocally that the cells detected by antibodies for epithelial keratin expression are really tumor cells. Thus, additional biomarkers have been studied to further characterize those epithelia-like cells. By combining staining of epithelial keratins with staining of PSA and fluorescence in situ hybridization (FISH), it has been shown that epithelial cells in BM aspirates that have been collected immediately after radical prostatectomy or cryosurgical ablation do really express PSA and are predominantly cytogenetically aberrant [97]. Several later published studies have confirmed tumor-specific characteristics in these epithelial-like cells supporting their designation as DTCs [98–100]. Nevertheless, some of the detected cells in the BM may still represent contaminating, epithelial keratin-expressing cells from the skin or endothelium [100].

In the following decade, several studies have validated the prognostic significance of DTCs in the BM of early-stage prostate cancer patients in different clinical settings. For prostate cancer patients who received radiotherapy, the detection of DTCs in BM at diagnosis was associated with the histological differentiation of the primary tumor and an increased risk of developing distant metastases during a 7-year follow-up [101]. Similar results were obtained for patients that received hormone therapy followed by radical prostatectomy. Here, the presence of DTCs in BM was a significant prognostic factor with respect to poor PSA progression-free survival and an independent predictor of biochemical recurrence in a multivariable analysis [102]. In addition, in the setting of radical prostatectomy without neo-adjuvant therapy, the detection of DTC prior to surgery was an independent predictor of recurrence [103]. This finding was confirmed in another study, showing that patients in whom DTCs were detected preoperatively were more likely to relapse within the first 2 years after surgery, but in this study, the detection of DTCs in the postoperative setting was not correlated with biochemical recurrence or the development of metastatic disease [104]. One more study addressed the question, whether the detection of DTCs in BM of prostate cancer patients before or after treatment for prostate cancer could be used as a prognostic marker for recurrence. In this study, only preoperative DTC status showed up as statistically independent parameter for survival in the multivariate analysis [105]. Nevertheless, also conflicting results have been published. In a single-center study, analyzing a cohort of patients with increased risk for disease recurrence, the detection of DTCs at the time of prostatectomy was not correlated to the clinical outcome [106]. This discrepancy might be due to technical variations in the assays applied or differences in study cohorts. However, to date, the analysis of BM aspirates at time of surgery is not recommended as a standard procedure in patients with clinically localized PC.

In summary, these results imply that the presence of DTC in the BM of prostate cancer patients is an indicator of MRD. Based on these findings several clinical applications have been proposed. Intended usages for DTCs would be as a biomarker of prognosis that predicts for disease recurrence after surgery or to identify patients that would benefit from anti-proliferative therapy in the perioperative or postoperative period [107]. As postoperative detection of DTCs does not always predict for poor overall survival [104, 106], it is feasible that DTCs in the BM of prostate cancer patients can remain in a state of dormancy for extended periods. During the last decade, the interest in the detection of DTCs has declined and also the latest mechanistically studies on mechanisms that control tumor dormancy of prostate derived DTCs, lacked the opportunity for validating results in BM samples collected from real tumor patients. This is possibly because BM sampling is an invasive procedure not integrated into clinical routine. In recent years and maybe due to the declining interest in DTC detection, there have not been any technical advancements for the enrichment and detection of DTCs, too. Nevertheless, there is one report describing the evaluation across multiple CTC analysis platforms revealed that these technologies are nonspecific in BM and, thus, not suitable for DTC detection [108]. However, we propose that the detection and molecular characterization of cancer cells in metastasis-prone environment provide complementary information to other biomarkers, and we encourage other scientist to include DTC analysis in future research studies.

### Monitoring MRD in prostate cancer patients using blood-based biomarkers

#### Circulating tumor cells (CTCs)

Besides other biomarkers, CTCs have been most intensively analyzed in prostate cancer [109] and the assessment of CTCs or tumor cell-derived products in the circulation, such as cell-free nucleic acids or extracellular vesicles bear also the potential to identify prostate cancer patients with MRD. In prostate cancer, it has been shown that higher numbers of CTCs are not simply a matter of an increasing disease burden, but also an intrinsic property of the tumor [110]. Nevertheless, there is a clear correlation between the number of detectable CTCs and the tumor stage. Thus, CTC detection in prostate cancer is best suited and validated for monitoring metastatic prostate cancer patient with castration-resistant disease, in whom CTCs are more often detected and at higher numbers [109–112]. In contrast, utility of CTC detection in early-stage prostate cancer patients and relevance of blood-based assays to monitor MRD in non-metastatic patients remains less conclusive due to irregular and low CTC counts. In addition, CTC enumeration does not correlate with other clinic–pathological parameters in these patients [109]. However, recently, it has been shown that CTC enumeration by CellSearch analysis before a salvage lymph node dissection can indicate spread of tumor cells via the blood and systemic tumor disease. Suggesting that CTC-positive patients seem to have worse pathological and short-term oncological and will probably not benefit of lymph node dissection [113]. In addition, the in vivo CellCollector has successfully been applied to detect CTCs before and after radiotherapy, suggesting that CTC is a suitable biomarker in high-risk non-metastatic prostate cancer patients [114].

Collectively, highly sensitive CTC detection and characterization methods are required to identify prostate cancer patients with MRD for patients with localized disease. In order to enhance the value of CTC detection in early-stage patients with MRD, combined and complementary CTC isolation and detection techniques, like CellSearch, CellCollector, and EPISPOT assays have successfully been applied [115–117] (Table 1). Another possibility to enhance the sensitivity for MRD detection is the implementation of prostate-specific markers in liquid biopsies for the identification of rare prostate cancer cells. In theory, in order to yield a high sensitivity such prostate-specific markers shall be stably expressed throughout the entire disease progression and shall not be expressed on non-prostate-cancer cells in the sample [118]. In this context, multimarker RNA profiling of individual CTCs offers the opportunity to analyze gene expression of multiple markers simultaneously [119]. Nevertheless, capture of the extremely rare CTC in early-stage patients remains the bottleneck for implementing prostate-specific markers in liquid biopsies approaches.

Only few studies compared side by side the clinical relevance of CTCs and DTCs in the same PC patients. Murray et al. have simultaneously banalyzed CTCs and DTCs to identify MRD patients in a cohort of patients with classified pathologically organ-confined prostate cancer (pT2) treated by radical prostatectomy. Patients were classified as (i) MRD negative (CTC and DTC negative), (ii) micro-metastasis positive, and (iii) CTC positive. After 10 years of follow-up, a significantly increased risk for biochemical recurrence has only been found for CTC-positive patients compared to the MRD negative group, whereas no increased risk has been detected for DTC positive patients [120]. These results imply that CTC detection is superior to DTC detection for the identification of MRD patients, but these results need to be confirmed in larger studies using state of the art technologies.

#### Circulating tumor DNA (ctDNA)

Besides CTCs, other tumor-derived biomarkers, like ctDNA, have been suggested for blood-based analysis for the identification of prostate cancer patients [121]. In this context, it has been reported that in particular hypermethylation the *ZNF660* promotor can be potential used as blood-based biomarker for the stratification of low/intermediate-grade cases into indolent or more aggressive subtypes [122]. Exosomes also offer a potential biomarker content that could be used alone or in combination with other types of liquid biopsies [123] but to our knowledge successful applications for MRD detection in prostate cancer have not been reported, so far. However, any biomarker for MRD detection in prostate cancer needs to compete with serum PSA testing, which is an excellent marker in aspects such of monitoring treatment response and/or tumor relapse. Like for all other cancer entities, low allele fractions at post-treatment time points as well as interference of technical and biological background are hampering reliable results from ctDNA analysis for MRD detection [124] and there is an ongoing debate whether ctDNA detection could really measure up with PSA testing for MRD detection in prostate cancer [125]. Nevertheless, we believe that complementary methods, analyzing different biomarker is the best strategy to enhance MRD detection and the complementation but not the replacement of PSA testing should be the goal of current research efforts.

## Melanoma

### Clinical relevance of MRD in patients with melanoma

Malignant melanoma is the 13th most common cancer in men and the 15th most common cancer in women. The absolute number of incident melanoma cases increased continuously since 1999 [126, 127]. Because of primary tumor heterogeneity and progressive clonal divergence resulting in the growth of more aggressive tumor populations the majority of early-stage, non-metastatic melanomas will experience recurrence following a variable disease-free interval and progression to metastatic melanoma and ultimately death. The 5- to 10-year overall survival (OS) rates for clinical stage IIIB, IIIC, and IIID are 83–77%, 69–60%, and 32–24%, while in Stage IIB/IIC approximately 11% (low risk)–28% (high risk) of patients without adjuvant therapy experience relapse after 18 months [128]. Melanomas are highly immunogenic tumors, as seen by the naturally occurring high level of T cell infiltration and, in some patients, spontaneous tumor regression. Therapeutic strategies to eradicate dormant cells by impairing important survival pathways or mechanisms that mediate therapy resistance are promising. By inhibiting the lymphocyte activation gene 3 (LAG-3), the glucocorticoid-induced TNF receptor (GITR), and the T cell immune receptor with immunoglobulin and ITIM domain (TIGIT), the T cell antitumor response is improved, which leads to increased T cell effector and NK cell proliferation, resulting in a more efficient elimination of dormant tumor cells [2]. With a minimal risk of overall toxicity, promising techniques combine antiangiogenic treatment or cancer vaccines with immunotherapy to activate tumor-specific immune responses with long-term memory to prevent recurrence or metastasis [129–131]. Many of the most interesting new drugs relate to immune-mediated quiescence. Similar to prostate cancer, there are commercially available tumor markers in melanoma patients such as S100β, LDH, the protein “melanoma inhibitory activity” (MIA), CRO, PD-L1, IL-8, TIL, osteopontin, and YKL-40. However, their clinical utility is limited as many of these markers are associated with other biological processes. Even if the value of S100-β as a prognostic marker in melanoma is low, there is still a correlation with the patient’s tumor burden and thus an association with overall survival of tumor-bearing metastatic melanoma patients. However, for tumor-free patients, for example, patients after surgical removal of lymph node metastases (Stage II/III), there is no correlation between S100-β serum concentration and overall or recurrence-free survival [132]. The success of targeted therapy (TT) and immunotherapy (ICI) in patients with metastatic melanoma led to the development of adjuvant therapy for high-risk melanoma. These therapies have now become the standard of care. Nine large randomized controlled trials of immune checkpoint inhibitors and targeted therapies in adjuvant treatment have shown improved recurrence-free survival compared with placebo or an active control group. However, following putative curative therapy without detectable tumor, recurrence can occur in approximately one-quarter of patients within the first five years after initial cancer treatment [133, 134]. Due to toxicities of adjuvant therapies, one goal in Stage II patients with primary surgical treatment is to identify patients at high risk of relapse. Thus, an optimal balance between insufficient treatments vs. overtreatment should be found. To address this issue, there are several biomarker-based gene expression profiling approaches for biomarker-based risk classification of patients at high risk for disease recurrence (MelaGenic, Skyline DX, Decision DX). In addition, recent trials determine mutational und molecular pathological relapse pattern of adjuvant therapy [135, 136]. One strategy currently under investigation is combining BRAF/MEK inhibitors with ICI. This combination strategy combines the hope for both a fast and lasting response to therapy. Due to the higher toxicity of triple combinations, and failed (COMBI-i) or disappointing (TRILOGY) trials, a sequencing strategy rather than a simultaneous triplet is thought to successfully combine the advantages of both treatment regimes in order to achieve superior response rates and increased duration of response (SECOMBIT, ImmunoCobiVem) [137, 138]. Regarding the many immunomodulatory therapeutics, currently being tested in numerous trials a tool for individual therapy decision making and recurrence prevention is of even greater importance. To fully understand the monitoring of tumor dormancy and early detection of disease recurrence, it is crucial to study the interactions between cancer cells and the surrounding microenvironment.

### Monitoring MRD in melanoma patients by detection and characterization of DTCs

Only a small part of CTCs can successfully arrive at a distant organ and become DTCs. Forty percent of melanoma patients develop distant metastases at five or more years after curative surgery, and frequent manifestations of melanoma without an identified primary lesion may reflect the tendency of melanoma cells to spread from indolent sites such as BM [139]. DTCs are found in 57.4% of skin melanoma cases and in as many as 28.6% of stage I cases, which confirms the aggressive course even of localized disease. Observations of hematogenous metastases from melanoma after 10 [24] or even 40 [25] years after removal of the primary tumor and frequent melanoma manifestations without cancer of unknown primary show the tendency of circulating melanoma cells (CMCs) to disseminate in the attractive metastatic niches, e.g., into the BM. gp100—HMB-45 has been used as marker of melanoma cells to identify DTCs in BM [28]. Examination of the BM of 47 melanoma patients revealed significant changes in BM hematopoiesis occurring in the presence of DTCs. Significant differences in the groups with the presence of DTCs (DTCs +) and their absence (DTC-) were found for blast cells, total granulocyte cell content and erythroid germ indicators, suggesting that myelo- and erythropoiesis are involved in the tumor process in the body and possibly react to the presence of DTCs, which can lead to a reorganization of the microenvironment [140]. Furthermore, using RET transgenic mouse melanoma model, dormant tumor cells accumulated in the BM were found to be co-localized with memory CD8 + T cells and displayed an aberrant expression of p16, p27, Ki67, and PCNA proteins, suggesting their dormant phenotype [141]. Although great advances have been made in CMCs, CTC isolation, and analysis, the clinical utility of melanoma CMCs need still to be investigated. Regarding the challenges that appear intrinsic to CMCs (i.e., rarity and heterogeneity) and due to a lack of standardization for CMC detection further investigations on CMC phenotypes, their prognostic potential as well as their differential pharmacodynamic responses to treatment is needed.

### Monitoring MRD in melanoma patients using blood-based biomarkers

#### Circulating tumor DNA (ctDNA)

The analysis of circulating tumor components in the blood such as ctDNA or CTCs shows promising potential and is used and investigated as a biomarker in many studies of other tumor entities like breast and prostate cancer [142]. Although most patients with early stages of melanoma exhibit a substantial gap between onset of primary and metastatic tumors, signaling mechanisms implicated in the period of metastatic latency remain unclear. As patients are rarely re-biopsied, detection in blood might be advantageous by enabling a comprehensive assessment of tumor mutational status in real time [143, 144]. Significant advances in ultrasensitive detection and characterization of CTCs and ctDNA allow now identifying MRD in an individual melanoma patient at a time point when there are still no clinical or radiological signs of distant metastases [94]. Váraljai et al. postulate that increasing ctDNA levels predicted disease progression significantly earlier than routine radiologic scans, with a mean lead time of 3.5 months. Current studies indicate that ctDNA concentration assessed during TT or immune checkpoint inhibition in melanoma patients seem to be a strong prognostic biomarker for advanced and adjuvant staged melanoma patients and can be used to predict response to treatment, recurrence, and resistance [145–147]. Recent studies have shown that the detection of ctDNA before surgery correlates with the aggressiveness of the disease. There is a high risk of recurrence after complete surgery for stage IIB, IIC after proper staging, thus including melanoma patients with a thick primary but negative sentinel node biopsy. The detection of ctDNA was an independent predictor of survival with a higher significance in patients with stage IIID compared to IIIC, and it was associated with a larger nodal melanoma deposit, a higher number of lymph node involvement and an increase in LDH levels [148]. As a diagnostically important biomarker for melanoma, the detection of the BRAFV600E aberration at the DNA and protein level in liquid biopsies confers an attractive option. Through identifying quiescent melanoma cells in intravascular niches of various metastatic organs, evidence of endothelial transdifferentiation (EndT) in BRAFV600E-metastatic biopsies from the human lung, brain, and small intestine reveals a tumor vascularization pattern that may contribute as a potential therapeutic target to induce quiescence in metastatic organs of melanoma [149]. Furthermore, it has been shown that the tumorigenic potential of a cancer cell correlates to its differentiation status and melanoma cells can acquire a metastable pluripotent state independent of BRAF or NRAS mutations [150]. Blood-based testing compared favorably with standard-of-care tissue-based BRAF mutation testing. Importantly, blood-based BRAF testing correlated with clinical outcome and appears to be therefore suitable for future interventional trials [147]. In addition to the qualitative and quantitative detection of the presence of specific mutations such as BRAF, KRAS, and NRAS, ctDNA clearance seems to gain importance as well. Baseline and postoperative ctDNA detection in two independent prospective cohorts identified stage III melanoma patients at highest risk of relapse and has potential to inform adjuvant therapy decisions. No relapse was observed in treated patients who did not have ctDNA at any time point. A different scenario was observed in the cohort of untreated patients with detectable postoperative ctDNA, where the relapse rate was 100% highlighting the potential of ctDNA as a predictive biomarker of relapse and survival [151]. Similarly, Lee et al. examined ctDNA concentration in melanoma Stage IV patients during therapy and showed that in the cohorts of patients in which ctDNA was not depleted at any time point or in which ctDNA clearance occurred during therapy, there was a significant survival advantage compared with the group that had a steady ctDNA concentration during therapy [152] (Table 1). However, ctDNA was not able to predict or monitor intracranial disease activity [153]. Despite the high potential of ctDNA as a prognostic biomarker, the standardization of a highly sensitive and reproducible methodology is warranted before translating liquid biopsy in clinical practice.

#### Circulating tumor cells (CTCs)

CTCs are cancer cells circulating in the peripheral blood shed from either the primary tumor or its metastatic sides. CTC analyses enable comprehensive assessment at the DNA, RNA, and protein levels [3], yet technical challenges to detecting and capturing CTCs must be addressed. Investigating the heterogeneity of tumors within a patient through CTC analyses are an important mechanism to uncover MRD and recurrence after or under ongoing treatment [33]. However, detection of CTCs in melanoma patients has been challenging in recent decades due to the remarkable phenotypic plasticity of melanoma cells. Despite the high phenotypic and molecular heterogeneity of melanoma CTCs, the EMT process is believed to play an important role in CTC dissemination. CTCs share the mutational profile with primary cells, an intermediate EMT phenotype, and high expression of the immunosuppressive factors. EMT and acquisition of stem-like properties can dictate tumor cell quiescent and or their proliferative fate [154]. A subclonal CTC population exhibited stem cell properties as high aldehyde dehydrogenase 1 activity, melanosphere-forming ability, and expression of major stemness transcription factors. Xenograft experiments confirmed the CTC ability to generate melanoma in vivo and revealed enhanced metastatic propensity [155]. Commonly used CTC markers include tyrosinase, Melan-A/MART-1, MAGE3, MCAM, gp-100, MITF, and *Gal*Nac-T, which have specificities ranging from 85 to 100% but sensitivities from 6 to 95%. Detection of tyrosinase mRNA in peripheral blood may be of similar importance for the clinical course of melanoma as the detection of micrometastatic disease in the sentinel lymph node. Whether a combination of these two factors leads to a better definition of the prognosis of melanoma patients is under investigation in current studies [156]. Multimarker-derived CTC scores could serve as viable tools for prognostication and treatment response monitoring in patients with metastatic melanoma. CTC detection using a combination of immunocytochemistry and transcript analyses of five genes by RT-PCR and 19 genes by droplet digital PCR (ddPCR) was associated with shorter overall and progression-free survival. In addition. Lucci et al. determined that ≥ 1 CTC was independently associated with melanoma relapse, suggesting that CTC assessment may be useful to identify patients at risk for relapse who could derive benefit from adjuvant therapy [157]. CTC isolation from peripheral blood using a high-density dielectrophoretic microwell array, followed by labeling with melanoma-specific markers (MART-1 and/or gp100) and a leukocyte marker (CD45) of a few stages 0-III melanoma patients detected CTCs even in patients with early disease (stage 0 and I). By selecting three informative biomarkers (MART1, MAGE-A3, and GalNAc-T), Koyanagi et al. demonstrated that two or more positive biomarkers were significantly associated with worse distant metastasis disease-free survival and reduced recurrence-free survival [158]. Interestingly, the number of CTCs seems to reflect patients’ responses to BRAF/MEK inhibitor treatment indicating a usefulness of CTC analysis for monitoring response to TT [159]. CTC scores correlated with plasma ctDNA concentrations and had similar pharmacodynamics changes upon treatment initiation. The outcomes of patients with melanoma who have sentinel lymph node (SLN) metastases can be highly variable. Detection of CTCs in patients with melanoma diagnosed with SLN metastases Stage III individual CTC biomarker detection ranged from 13.4 to 17.5%. First-time evidence provide that the asymptomatic progression of metastatic melanoma can be recapitulated in vivo using patient-isolated CTCs. Implantation of Lin- population in NSG mice (CTC-derived xenografts, i.e., CDX), and subsequent transcriptomic analysis of ex vivo BM-resident tumor cells (BMRTC) versus CTC identified protein ubiquitination as a significant regulatory pathway of BMRTC signaling. The authors assume targeting BM-resident tumor cells through pharmacological inhibition of USP7 as a possible therapeutic strategy [160].

## Conclusion

Cancer dormancy tends to shift into lethal recurrence, posing severe challenges to clinical treatment. Blood based biomarker analysis offer a unique opportunity to determine—specifically from DTCs, CTCs, or ctDNA—whether this circulating material demonstrates full metastatic potential, associated with rapid disease recurrence and death, or, whether it is a terminally differentiated tumor mass with no clinical relevance or a dormant cancer cell that may be associated with long-term adverse outcomes [161]. Depending on the scenario, different treatment options are needed. Over the past decade, molecular phenotyping and genotyping of CTCs and DTCs have taken a first step toward this goal. Validation of previous findings to detect mechanisms of tumor dormancy in DTCs in BM samples from patients remains challenging due to the current still invasive procedure. Nevertheless, the detection and molecular characterization of cancer cells in an environment prone to metastasis provide complementary information to other biomarkers [162, 163].

Tumor heterogeneity is a hallmark of solid tumors and has an impact on the classification, diagnosis, and future treatment of cancer. Assessment of ctDNA and CTCs can be also used to encompass intrapatient and interpatient tumor heterogeneity in cancer patients. Furthermore, analysis of ctDNA offers a promising tool for adequate therapy monitoring and for risk profiling of relapse especially under therapy, which plays an important role in tumor dormancy as an extrinsic factor. Regarding the challenge of enrichment and detection of circulating blood-based biomarker, e.g., short survival time of CTCs in the bloodstream or low concentration of ctDNA in early cancer stages, liquid biopsy assays need to be more standardized. Regarding this international consortium such as the European Liquid Biopsy Society (ELBS, www.elbs.eu), it can play an important role [164]. Composite biomarker panels need to be tested in clinical trials with established endpoints to demonstrate clinical validity and utility, which will be critical for the introduction of LB into clinical practice. Preclinical research will enable the discovery of new pathways responsible for the survival of quiescent cancer cells and the identification of mechanisms that cause the transition from quiescent to active disease. The development of targeted molecular therapies aimed at eliminating dormant residual tumor cells or maintaining them in a quiescent state is a highly attractive approach to prevent late tumor recurrence. In addition, experimental studies need to gain more knowledge about LB marker biology, which in turn can be applied to the patient to improve the clinical use of LB analytes. The insights into tumor dormancy and its impact on MRD and eventual metastasis described in this review have the potential to advance personalized medicine significantly. Overall, risk stratification by genomic analysis of primary tumors of patients, combined with a high-frequent MRD detection by DTCs, ctDNA, and CTCs enables future clinicians to prevent overt metastasis formation and paves the way for better clinical management.

## Abbreviations

AJCC: American Joint Committee on Cancer
AUC: Area under the curve
BM: Bone marrow
BPH: Benign prostatic hypertrophy
CMC: Circulating melanoma cells
CSC: Cancer stem cell
CTC: Circulating tumor cells
ctDNA: Circulating tumor DNA
CTX: Chemotherapy
ddPCR: Droplet digital PCR
DFS: Disease-free survival
DTC: Disseminated tumor cell
EpCAM: Epithelial cell adhesion molecule
EPISPOT: Epithelial immunospot
EMT: Epithelial to mesenchymal transition
ER: Estrogen receptor
ICC: Immunocytochemistry
ICI: Immune checkpoint inhibition
LB: Liquid biopsy
LRFS: Locoregional relapse-free survival
MRD: Minimal residual disease
NAT: Neoadjuvant therapy
NCDB: National Cancer Database
OS: Overall survival
Pat: Patients
PC: Prostate cancer
PR: Progesterone receptor
PSA: Prostate-specific antigen
RT: Radiation therapy
TNBC: Triplet-negative breast cancer
TR: Time ratio
TT: Targeted therapy
